# Effective separation and simultaneous analysis of anabolic androgenic steroids (AAS) in their pharmaceutical formulations by a validated TLC-densitometry method

**DOI:** 10.1186/1752-153X-6-54

**Published:** 2012-06-15

**Authors:** Syed Ghulam Musharraf, Umair Gulzar

**Affiliations:** 1 Dr. Panjwani Center for Molecular Medicine and Drug Research, University of Karachi, Karachi, 75270, Pakistan; 2 H.E.J. Research Institute of Chemistry, International Center for Chemical and Biological Sciences (ICCBS), University of Karachi, Karachi, 75270, Pakistan

**Keywords:** Testosterone derivatives, TLC-densitometry, Testosterone propionate, Testosterone phenyl propionate, Testosterone isocaproate, Testosterone deaconate

## Abstract

**Background:**

Anabolic androgenic steroids (AAS) are widely misused for the enhancement of performance in sports. Several drugs are available that contain different combinations or individual steroids in different dosage form. This paper describes a TLC densitometric method for simultaneous determination of four AAS of testosterone derivatives including testosterone propionate (TP), testosterone phenyl propionate (TPP), testosterone isocaproate (TI) and testosterone deaconate (TD) in their pharmaceutical products.

**Results:**

Separation was carried out on Al based TLC plates, pre-coated with silica gel 60F-254 using hexane and ethyl acetate (8.5:1.5, v/v). Spots at *R*_f_ 0.31 ± 0.01, 0.34 ± 0.01, 0.40 ± 0.01 and 0.45 ± 0.02 were recognized as TPP, TP, TI and TD, respectively. Quantitative analysis was done by densitometric measurements at *λ*_max_ 251 nm for all derivatives. The developed method was validated as per ICH guidelines. Method was found linear over the concentration range of 200–1200 ng/spot with the correlation coefficient of 0.995, 0.993, 0.995 and 0.996 for TP, TPP, TI, TD, respectively. Limit of detection for all derivatives were in the range of 16.7-22.3 ng/spot while limit of quantitation were found to be in the range of 55.7-70.9 ng/spot.

**Conclusions:**

The developed TLC method can be applied for the simultaneous routine analysis of testosterone derivatives in their individual and combined pharmaceutical formulations.

## Background

Anabolic androgenic steroids (AAS) are mainly a group of natural or synthetic compounds that are chemically similar to the actions of androgynous testosterone, which is primarily a natural male hormone responsible for androgenic and anabolic effects observed during male adolescence and adulthood, and are modified to improve the anabolic effect of testosterone rather than its androgenic effect [1]. In addition to their medical uses, AAS have been widely misused by variety of athletes with the hope of improving their performance [2,3]. The use of AAS in sports has been banned since the mid-1970s but they are still the most misused class of drugs in sports. Moreover, steroid abuses have also become more and more prevalent outside sports.

Because of the short half-life of only one hour, exogenous intake of pure testosterone do not have any effect, since only 2% of oral intake reaches to the muscles. To slow down the metabolism and receive better effect, the testosterone molecule has been modified at its 17-position which creates stronger anabolic effect and weaker androgen effect [4]. Among these, TP, TPP, TI and TD are some of the ester derivatives of testosterone synthesized with the goal of prolonging the biological activity of parent molecule. Predominantly, they are administrated as intramuscular injection and are commercially available with different brand names like Sustanon, Testolic, Deca-Durabolin and Adrex. These injections come frequently from the black market and some are only for the veterinary use. Total dosages used are often above therapeutic level and some times more than one derivative is present in one injection in different proportion. Therefore, it’s an important need to develop a method for the the quality assessment of pharamacutical drugs commercially available in the market.

Thin-layer chromatography (TLC) continues to be an important method for the determination of steroids because of its simultaneous detection procedures, low detection limit up to nano gram range, and low cost has increased its importance in quantitative methods of analysis [5-8]. Szepesi and Gazdag have contributed significantly on the TLC analysis of steroids [9]. It included sample preparation as well as stationary-phase and mobile-phase systems which are useful for the separation of steroidal pharmaceutics [10]. Dreassi *et al.*[11] have also reviewed the application of TLC to steroids in pharmaceutical analysis while Jain *et al.* has provided some information on the analysis of steroidal hormones by TLC in clinical chemistry [12]. Recently, Bhawani has given a detailed review on the methods for the analysis from 1990 to 2009 [13]. To the best of our knowledge, no work has been published related to the analysis of testosterone and its ester derivative using TLC-densitometry. In continuation of our studies on TLC-densitometry method development of pharmaceutical drugs [14], this work describes analysis of testosterone and its most common ester derivatives in the pharmaceutical formulations.

## Experimental

### Materials

Testosterone derivative standards, TP, TPP, TI and TD were complementarily provided by M/S Hilton Pharma (Pvt.) Ltd, Karachi, Pakistan. Pharmaceutical products, injections of different testosterone derivatives including Testolic (S-1) and Sustanon (S-2) were purchased from a pharmacy shop in Karachi, Pakistan. Methanol and dichloromethane of analytical grade were purchased from the Fisher Scientific (UK).

### Preparation of standard solutions

Stock solution was prepared by accurately weighing 5 mg of each standard and dissolved in 10 mL dichloromethane. Stock solution was further diluted in dichloromethane to obtain working standard solutions of various concentrations and stored at 4°C until use. Different microliters of each working standard were spotted on the TLC plate for the six standard levels including 200, 400, 600, 800, 1000, and 1200 ng spot^−1^ for the calibration curve in triplicate and subjected to plate development, followed by densitometric analysis at λ_max_ 251 nm. This practice was repeated six times to get an average standard calibration curve at concentration range 200–1200 ng/spot.

### Preparation and analysis of pharmaceutical products

Pharmaceutical samples were prepared by dissolving the 1 mL injection of steroid suspension (made in coconut oil) in 50 mL of dichloromethane and stored at 4°C until use. 10 μL from each sample solution was spotted on the TLC plate in triplicate and subjected to plate development, followed by densitometric analysis.

### Instrumentation and chromatographic condition

A CAMAG system, equipped with automatic TLC sampler (LINOMAT 5), TLC scanner 3 and integrated software of WinCats (version 1.2.3) was used for the analysis of testosterone derivatives. Precoated silica gel TLC-cards (60F-254, 20 cm × 10 cm, E. Merck, Darmstadt, Germany) was used. Samples and standards were spotted on TLC card in the form of bands of width 6 mm with a CAMAG 100 μL syringe using a CAMAG Linomat 5 auto sampler. A constant application rate of 0.1 μL s^-1^ was employed, and the space between two bands was 8 mm. Different mobile phases with varying ratios were tried to optimize *R*_f_ values of the compounds. Plate development was carried out in twin trough chamber (CAMAG, Muntenz, Switzerland) with optimized mobile phase (15 mL) of hexane and ethyl acetate (8.5:1.5, v/v) under unsaturation condition. Video densitometry was carried out with CAMAG Reprostar 3 and scanning was performed on CAMAG TLC Scanner III at 251 nm which operates in reflection absorbance mode by WinCat software. Deuterium lamp with range 190 to 400 nm was used as light source. Evaluation of the amount of the sample was obtained by using peak areas in linear regression.

### Method validation

The developed method was validated in term of precision, LOD & LOQ, specificity and recovery studies according to the ICH guidelines. Linearity was evaluated by determining six working standard solutions over concentration range of 200–1200 ng/spot. Peak area and concentration were subjected to the least square linear regression equation to calculate the regression data and correlation coefficients. The linearity of the standard calibration curve was tested by residual linearity test. Limit of detection (LOD) and limit of quantitation (LOQ) were determined by the spotting of blank methanol six times and developed according to the described chromatographic conditions. The signal to noise ratio 3:1 and 10:1 for LOD and LOQ, respectively was considered. Moreover, both were experimentally confirmed by diluting the known concentration of each standard until the average responses were approximately three or ten times to the standard deviation of the responses for six replicate determinations. Repeatability and reproducibility of the method were evaluated using inter-day and intra-day analysis with different analyst, respectively. Intra-day precision of the method was evaluated by spotting pharmaceutical samples three times while Inter-day precision of the proposed method was determined by repeating experiment at different days over a period of one week and results were statistically evaluated in terms of % R.S.D. Standard addition method was used to check the recovery of the proposed method. Pre analyzed pharmaceutical drugs were spiked with extra 50, 75 and 100% of each standard of testosterone derivatives and then extracted with dichloromethane and analyzed using proposed method. The analysis of spiked samples was repeated three times. To verify the specificity of the developed method, standard and samples were analyzed simultaneously. The peaks of each standard in samples were confirmed by comparing the *R*_f_ and spectra of the peaks of samples with that of standard. The peak purity of each standard in samples was assessed by comparing the spectra of standard and samples at three different positions, peak start, peak apex and peak end positions. In order to check the robustness, following parameters were deliberately changed at three different concentration levels (300, 500 and 800 ng of each standard), scanning wavelength (λ_max_ ± 2nm), mobile phase volume (15 ± 2 mL), and time variation (30 minutes) before chromatographic process, were studied and effects on the results were examined.

## Results and discussion

### Development of optimum mobile phase

**Figure 1:**
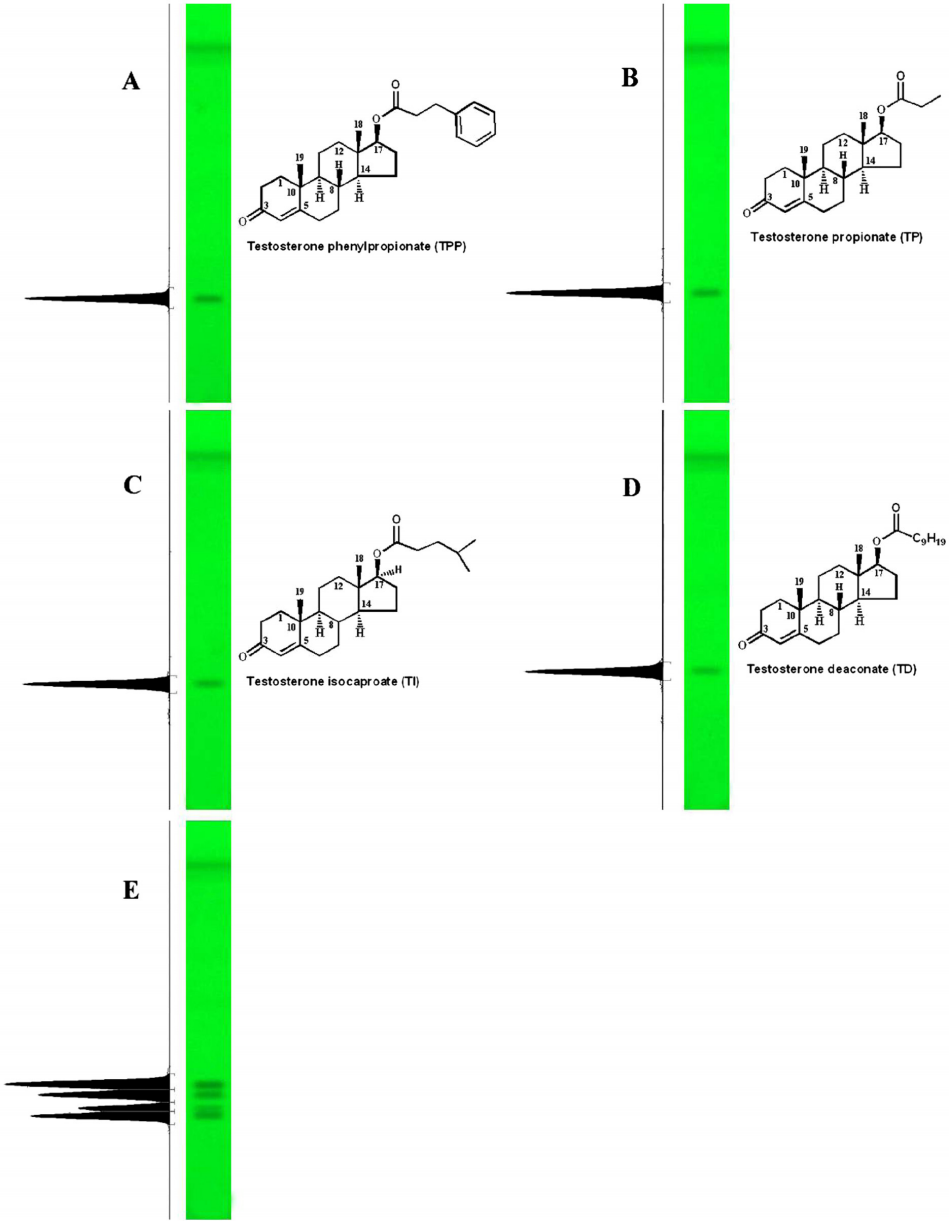
** Videodensitometries and UV chromatograms of standards at λ**_**max**_**251 nm.A** = Testosterone phenyl propionate (*R*_f_: 0.31 ± 0.01), **B** = Testosterone propionate (*R*_f_: 0.34 ± 0.01), **C** = Testosterone isocaproate (*R*_f_: 0.40 ± 0.01), **D** = Testosterone deaconate (*R*_f_: 0.45 ± 0.02) and **E** = mixture of standards.

A standard mixture of TP, TPP, TI, and TD were spotted on TLC plates for the development of optimum mobile phase. Various mixtures of solvent were used for the optimization of mobile phase selection and its composition (Table 1). Initially, hexane and ethyl acetate were kept constant while third solvent which were chloroform, dichloromethane and toluene varied in different composition. However, these combinations provided good *R*_f_ values but lacking sufficient resolution. Some of them were even unable to separate the testosterone derivatives from each other. Similarly, toluene and ethyl acetate (9.6:0.4, v/v) gave low *R*_f_ value with poor resolution. Finally, hexane and ethyl acetate (8.5:1.5, v/v) provided acceptable *R*_f_ value at 0.31 ± 0.01, 0.34 ± 0.01, 0.40 ± 0.01 and 0.45 ± 0.02 for TPP, TP, TI and TD, respectively with best resolution (Figure 1) under unsaturation condition. However, saturated condition was also tried but found no significant difference. Moreover, comparison of TLC plates with HPTLC plates showed no significant difference in peak width and *R*_f_ values (Table 1). TLC plates of different companies including Macherey-Nagel and Merck were also compared in which TLC plates from Macherey-Nagel gave higher *R*_f_ values but resolution with MERCK was found to be better. Finally, TLC plates from Merck with hexane and ethyl acetate (8.5:1.5, v/v) as mobile phase were used for further validation exercise.

**Table 1:** ***R***_**f**_**values and peak widths of the compounds in different mobile phases**

**S.No**	**Solvent composition**	**Ratios (v/v)**	**Retention factor value (*R***_**f**_**)**				**Δ*R***_**f1**_	**Δ*R***_**f2**_	**Δ*R***_**f3**_	**Peak Widths (cm)**			
			**TPP**	**TP**	**TI**	**TD**				**TPP**	**TP**	**TI**	**TD**
1	Toluene-Ethyl acetate	9.6:0.4	0.26	0.3	0.31	0.36	0.04	0.01	0.05	0.04	0.02	0.04	0.05
2	Toluene-Ethyl acetate-chloroform	9.0:0.6:0.4	0.35	0.41	X	0.46	0.06	X	0.05	0.05	0.06	X	0.05
3	Hexane-chloroform-ethyl acetate	7.0:1.0:1.5	0.63	X	0.67	0.73	X	0.04	0.06	0.09	X	0.06	0.05
4	Hexane-chloroform-ethyl acetate	7.5:1.5:1.0	0.23	X	0.29	0.33	X	0.06	0.04	0.08	X	0.04	0.05
5	Hexane-chloroform-ethyl acetate	8.3:1.0:0.7	0.36	0.38	0.44	0.48	0.02	0.06	0.04	0.04	0.04	0.05	0.06
6	Hexane-chloroform-ethyl acetate	8.3:0.7:1.0	0.38	0.41	0.46	0.51	0.03	0.05	0.05	0.04	0.04	0.06	0.05
7	Hexane-dichloromethane-ethyl acetate	8.0:0.7:1.3	0.54	0.54	0.61	0.65	X	0.07	0.04	0.1	X	0.05	0.06
8	Hexane-dichloromethane-ethyl acetate	7.5:1.0:1.5	0.67	X	0.74	0.77	X	0.07	0.03	0.11	X	0.05	0.05
10	Hexane-Ethyl acetate (Macherey-Nagel)	8.5:1.5	0.40	0.43	0.48	0.52	0.03	0.05	0.04	0.04	0.04	0.04	0.04
11	Composition 10 (TLC Plates-Merck)	8.5:1.5	0.31	0.34	0.40	0.45	0.03	0.06	0.05	0.05	0.04	0.06	0.05
12	Composition 10 (HPTLC Plate)	8.5:1.5	0.29	0.31	0.35	0.37	0.02	0.04	0.02	0.04	0.03	0.03	0.04

X = Shows no separate peak detected, **Δ*R***_**f1 =**_ between TPP and TP, **Δ*R***_**f2 =**_ between TP and TI, **Δ*R***_**f3=**_ between TI and TD.

### Calibration curves

The regression data showed a good linear relationship over a concentration range of 200–1200 ng/spot (Table 2) for all testosterone derivatives and having r = 0.995, 0.993, 0.995, and 0.996 for TP, TPP, TI and TD, respectively. Moreover, linearity of standard calibration curves was also verified by residual linearity test. Linearity was evaluated by preparing a standard solution of concentration 0.1mg/mL and different volume of this solution was applied to get the required range of 200, 400, 600, 800, 1000 and 1200ng/spot. Each concentration was simultaneously spotted on aluminum TLC sheets in triplicate.

**Table 2:** Linear regression data for the calibration curves (n = 6)

**Codes**	**Linearity range (ng/spot)**	***r*value**	**%R.S.D**	**Slope**	**%R.S.D**	**Intercept**	**%R.S.D**	**LOD ng/spot**	**LOQ ng/spot**
TP	200-1200	0.995	0.002	6.11	0.061	1494.6	0.111	16.7	55.7
TPP	200-1200	0.993	0.002	5.73	0.116	1471.8	0.164	21.2	70.8
TI	200-1200	0.995	0.001	5.47	0.064	956.9	0.164	22.3	70.9
TD	200-1200	0.996	0.002	5.01	0.056	783.7	0.191	21.1	70.5

### Inter and intra-day analysis

Injection samples, S1 containing testosterone propionate (TP) and S2 containing testosterone propionate, testosterone phenyl propionate (TPP), testosterone isocaproate (TI) and testosterone deaconate (TD) with known amount were used to check the precision of the method. Repeatability and reproducibility of the method was determined using inter and intra-day analysis. % Relative standard deviation (R.S.D. %) for all testosterone derivatives in samples were found to be less than 2% which shows good precision of proposed method (Table 3) while % R.S.D. for analyst 1 and 2 was found to be <3.2% in all cases.

**Table 3:** Intra- and inter-day analysis (n = 3)

**Sample**	**Intra-day precision**			**Inter-day precision**		
	**S.D. in amount (ng)**	**R.S.D. %**	**S.E**	**S.D. in amount (ng)**	**R.S.D. %**	**S.E.**
**S-1** a) TP	1.17	0.35	0.67	1.38	0.41	0.80
**S-2** b) TP	2.29	0.54	1.32	2.50	0.56	1.44
c) TPP	1.96	1.06	1.13	2.33	1.22	1.34
d) TI	0.27	0.05	0.16	3.22	0.64	1.86
e) TD	0.28	0.04	0.16	1.75	0.22	1.01

### LOD and LOQ

Limit of detection (LOD) and quantitation (LOQ) with 3:1 and 10:1 signal to noise ratio, respectively were calculated using linear calibration curves for each compound. Limit of detections was found to be 16.7, 21.2, 22.3 and 21.1 ng/spot for TP, TPP, TI, and TD, respectively while limit of quantitation was found to be 55.7, 70.8, 70.9 and 70.5 ng/spot, respectively (Table 2).

### Recovery studies

Recovery studies were carried out to find out the extraction efficiency of the proposed method. Extraction efficiency and matrix effects were evaluated using standard addition method. Each sample, S1 and S2 were spiked with additional 50, 75, and 100% of the standard testosterone derivatives and analyzed by proposed method. % Recovery for TP, TPP, TI, and TD were found in the range of 96.4-104.54, 102.58-106.1, 101.28-105.93, and 97.82-102.86, respectively. The results at each level of concentration of each testosterone derivative are summarized in Table 4.

**Table 4:** Recovery studies (n = 3)

**Excess amount added to injection (%)**	**Theoretical content (ng/μL)**	**Average experimental contents (ng/μL)**	**Recovery (%)**
a) TP			
50	350	337.4	96.40
75	440	460	104.54
100	500	516.25	103.25
b) TPP			
50	675	683.64	103.05
75	790	810.38	102.58
100	900	954.9	106.1
c) TI			
50	675	683.64	101.28
75	790	806.51	102.09
100	900	953.37	105.93
d) TD			
50	1200	1173.84	97.82
75	1400	1440.04	102.86
100	1600	1603.04	100.19

### Specificity

The peak purity of standard and both samples of testosterone derivatives were determined by comparing their spectra at peak start, peak apex and peak end positions. Good correlation, r (start, middle) =0.999 and r (middle, end) = 0.9999 were observed by comparing the spectra of standards and samples (Additional file 1: Figure S1).

### Robustness

The standard deviation of % yield of three standard levels 300, 500 and 800 was estimated for each parameter, mean R.S.D. % was 1.16 (TP), 1.78 (TPP), 1.32 (TI) and 1.18 (TD) for varying mobile phase volume, 2.12 (TP), 1.37 (TPP), 1.15 (TI) and 2.10 (TD) for the effect of varying wavelength, and 2.21 (TP), 1.96 (TPP), 2.27 (TI) and 1.85 (TD) for varying time from chromatography to scanning. Overall, R.S.D % is reasonably low < 2.27% for all parameters of robustness which indicates the developed method is robust for the quantification of above mentioned drugs in their pharmaceutical formulations.

### Sample analysis

The validated method was used for the analysis of two different testosterone derivative injection formulations. Testolic contains only one of the derivatives while sustanon-250 contains all four derivative of testosterone in different amount as indicated in Table 5. The analysis of marketed formulations of Testolic showed drug content of 96.4 mg of TP while Sustanon 250 showed drug content of 27.15, 67.61, 82.16 and 131.75 mg for TP, TPP, TI, and TD, respectively. Video densitometry of the standards and marketed formulations of testolic and sustanon are shown in Additional file 1: Figure S2. The low R.S.D. % value indicated the suitability of this method for routine analysis of these derivatives in pharmaceutical dosage forms.

**Table 5:** Sample analysis (n = 3)

**Brand name (sample code)**	**Compounds detected**	**Label claim (mg/injection)**	**Amount detected (mg/injection)** **(Mean ± S.D.)**	**% R.S.D.**	**% Rec.**
Testolic (S-1)	TP	100	96.4 ± 0.53	0.56	96.4
Sustanon 250 (S-2)	TP	30	27.15 ± 0.26	0.98	90.5
	TPP	60	67.61 ± 0.41	0.62	112.6
	TI	60	82.16 ± 0.55	0.68	136.9
	TD	100	131.75 ± 1.97	1.50	131.7

## Conclusion

A TLC-densitometric method for the simultaneous analysis of four anabolic androgenic steroids (AAS) of testosterone congers including testosterone propionate (TP), testosterone phenyl propionate (TPP), testosterone isocaproate (TI) and testosterone deaconate (TD) in pharmaceutical formulations was developed. The method was validated as per the ICH guidelines. Statistical data showed that the method is reproducible and selective for the quantification of the target analytes and can be effectively used for routine analysis of testosterone derivatives in pharmaceutical dosage formulations.

## Competing interests

The authors declare that they have no competing interests.

## Authors’ contributions

SGM: Participated in the experimental design and method optimization. UG: Performed the experiments and wrote the manuscript. All authors read and approved the final manuscript.

## Supplementary Material

Additional file 1 **Figure S1.** Overlay spectra of testosterone standards and two samples (S-1 and S-2). **Figure S2.** Video densitometry of all standards and sample including testosterone propionate standard (Track # 1-3), injection sample S-1 (track # 4-6), testosterone phenyl propionate standard (Track # 7-9), injection sample S-2 (Track # 10-12), testosterone isocaproate standard (Track #13-15), testosterone deaconate (Track # 16-18). Click here for file
