# Supplementary material for: Effective separation and simultaneous analysis of anabolic androgenic steroids (AAS) in their pharmaceutical formulations by a validated TLC-densitometry method
Source: Chem Cent J. 2012 Jun 15;6:54. doi: 10.1186/1752-153X-6-54 (PMC3469343; doi:10.1186/1752-153X-6-54)
Supplement: Additional file 1 — Figure S1. Overlay spectra of testosterone standards and two samples (S-1 and S-2). Figure S2. Video densitometry of all standards and sample including testosterone propionate standard (Track # 1-3), injection sample S-1 (track # 4-6), testosterone phenyl propionate standard (Track # 7-9), injection sample S-2 (Track # 10-12), testosterone isocaproate standard (Track #13-15), testosterone deaconate (Track # 16-18). [file 1752-153X-6-54-S1.doc]

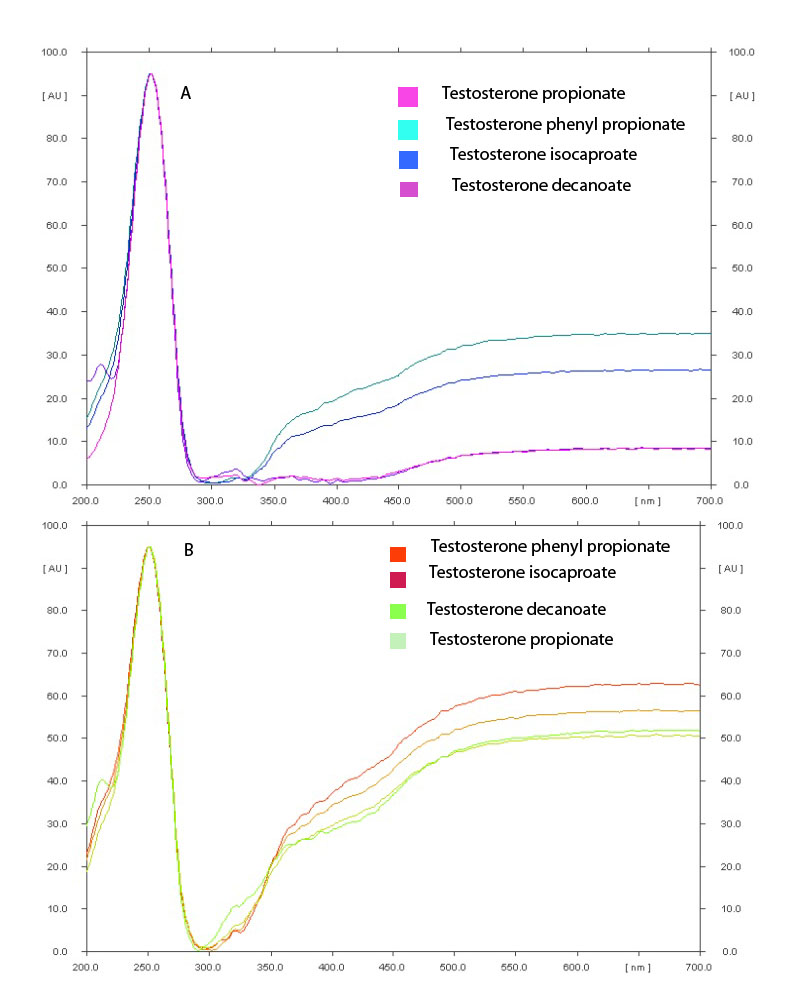


**Supplementary Figure 1**: Overlay spectra of testosterone standards and two samples (S-1 and S-2).


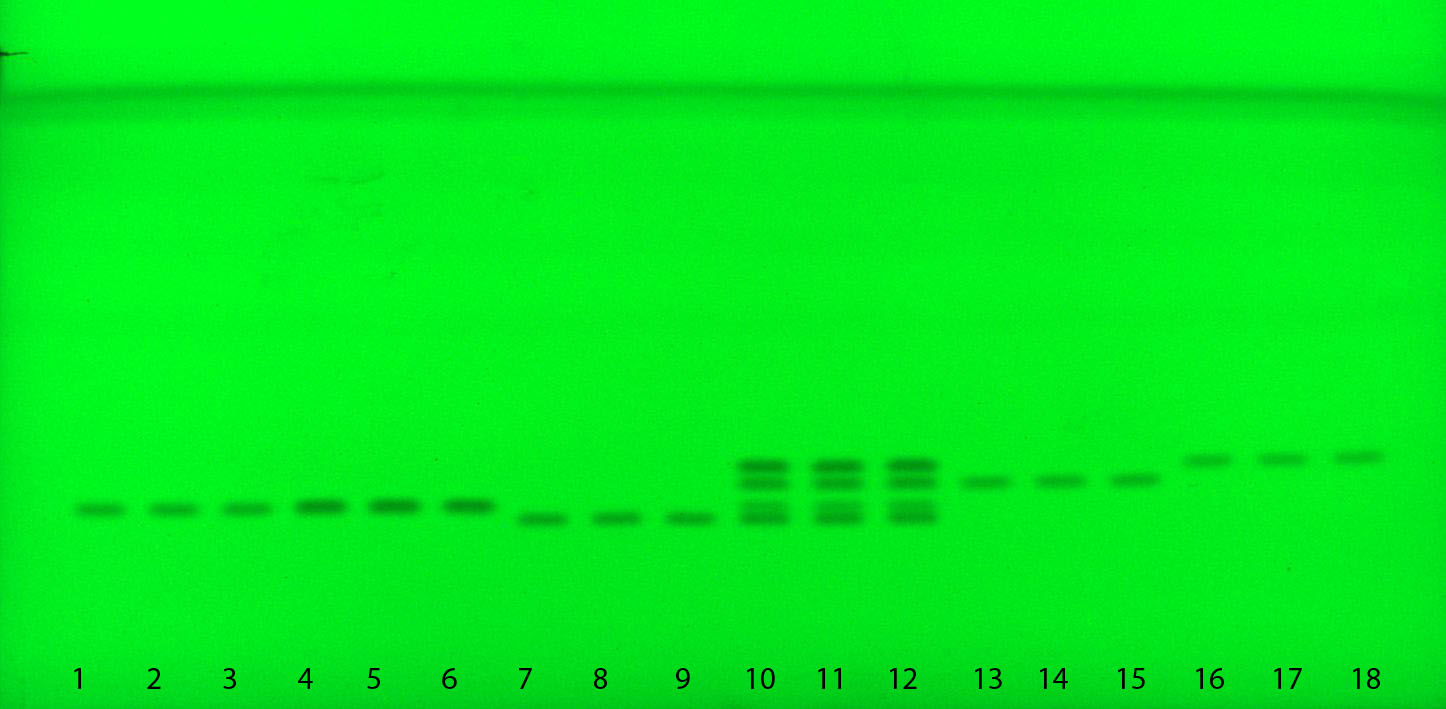


**Supplementary Figure 2**:Video densitometry of all standards and sample including testosterone propionate standard (Track # 1-3), injection sample S-1 (track # 4-6), testosterone phenyl propionate standard (Track # 7-9), injection sample S-2 (Track # 10-12), testosterone isocaproate standard (Track #13-15), testosterone deaconate (Track # 16-18).
